# Fluctuation relations for irreversible emergence of information

**DOI:** 10.1038/s41598-022-21729-9

**Published:** 2022-10-14

**Authors:** J. Ricardo Arias-Gonzalez

**Affiliations:** Centro de Tecnologías Físicas, Universitat Politècnica de València, Camino de Vera s/n, 46022 Valencia, Spain

**Keywords:** Statistical physics, Information theory and computation, Biological physics, DNA nanomachines

## Abstract

Information theory and Thermodynamics have developed closer in the last years, with a growing application palette in which the formal equivalence between the Shannon and Gibbs entropies is exploited. The main barrier to connect both disciplines is the fact that information does not imply a dynamics, whereas thermodynamic systems unfold with time, often away from equilibrium. Here, we analyze chain-like systems comprising linear sequences of physical objects carrying symbolic meaning. We show that, after defining a reading direction, both reversible and irreversible informations emerge naturally from the principle of microscopic reversibility in the evolution of the chains driven by a protocol. We find fluctuation equalities that relate entropy, the relevant concept in communication, and energy, the thermodynamically significant quantity, examined along sequences whose content evolves under writing and revision protocols. Our results are applicable to nanoscale chains, where information transfer is subject to thermal noise, and extendable to virtually any communication system.

## Introduction

Information management involves dynamic operations on a chain of physical objects carrying symbolic meaning. Such operations, technologically revolutionizing at atomic scales, comprise both physical manipulations of the objects, including mechanical maneuvering of or electromagnetic interactions with them, and purely configurational changes in the resulting sequence of symbols^1–4^. As a consequence, the chain-like system exchanges energy with the environment. Physical operations involve a thermodynamic analysis, whereas the computational aspects are often uniquely treated through information theory. However, information is also a physical entity amenable to be inspected through thermodynamic analysis^5^.

The links between information theory and statistical mechanics are expanding the thermodynamic view of a physical system and its environment^6–8^, with experimental verifications of the Landauer’s principle^9–11^ and evidence for the conversion of information to work^12–14^. A paradigm of the link between information and thermodynamics is the so-called central dogma of molecular biology, which describes the information flow from DNA to proteins. These ancient, natural systems, besides involving polymer chains, are nanoscopic and dynamic, often evolving away from equilibrium^15–20^, a feature that transcends to macroscopic scales in living beings^21^.

Information variations in a chain-like system are associated to energy transactions with the environment, which can take place reversibly or irreversibly, with a lower theoretical energy limit^22,23^. Fluctuations as a consequence of pure computations are on the order of the thermal level (i.e., similar to *kT*, being *k* the Boltzmann constant and *T* the absolute temperature), according to Landauer’s principle. Such energies are negligible at routine human scales but become significant when the size of the system is nanoscopic or smaller, because the work and heat it generates also compare with the thermal level. Small systems are based on nanostructures, including individual molecules and arrangements of atoms, such as biological and quantum systems.

Fluctuation theorems have appeared in recent years explaining quantitatively energy imbalances between forward and reverse pathways or between equilibrium and non-equilibrium processes^24,25^. They have been tested experimentally^26–28^, mostly in biomolecular systems analyzed on a one-by-one basis^29^. Most of these theorems establish relations among thermodynamic potentials for general systems, often with no specific insight into information theory. This theory, in turn, deals with spatially-indexed, 1-dimensional arrangements of symbols, which may not be necessarily associated to a time order. Recent generalizations separate the role of information and feedback control^30,31^, but still the interpretation of non-Markovianity, irreversibility and reversibility in terms of purely informational operations such as reading, writing and error correction^32,33^ remains obscured.

Here, we analyze energy exchanges associated to the symbolic management of a sequence of characters, without reference to the physical construction of the chain. Just by considering reversibility at the single sequence level and conservation laws, we next present two pairs of fluctuations equalities in the creation of information sequences, which use depends on energy exchange constraints. Our analysis integrates key information concepts, namely, reading, writing, proofreading and editing in the thermodynamic description of a string of symbols with information.

## Results and discussion

**Figure 1:**
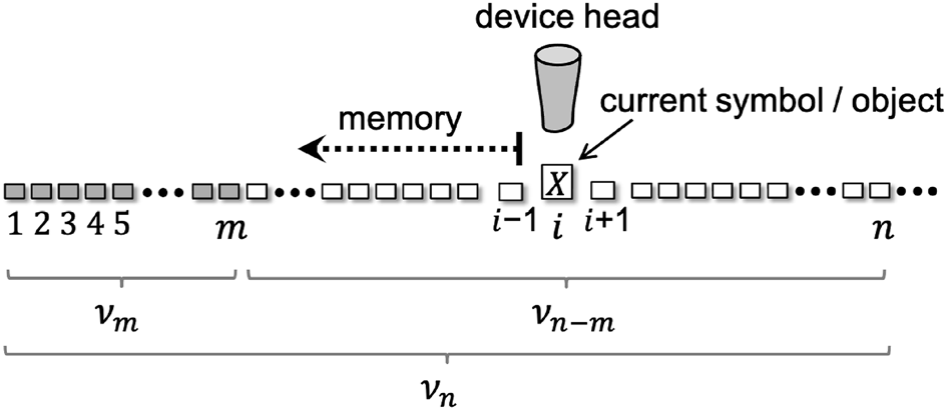
Sketch of the chain-like system, which represents a segment of a tape with a finite number of cells. At each computation step, *i*, a machine working on the tape follows instructions from a program, transition table or polymer template, considers the symbols already incorporated (from 1 to $i-1$) and its ‘head’ changes the value of the current cell. The segment sequence, $\nu _n$, is divided into an initial sequence $\nu _m$ and a remainder sequence $\nu _{n-m}$. The remainder segment may be initially inexistent or with a null sequence (meaningless values for the cells from $m+1$ to *n*).

The system under study is a chain made up of physical objects, represented by random variables $X_i=x_i$ ($x_i \in \mathcal{X}$, being $\mathcal{X}$ the alphabet of the symbols or domain of the discrete variables with cardinality $|\mathcal{X}|$, and $i=1,2,\ldots$ the linear sequence). A microstate of the chain-like system builds up as a single microscopic sequence of values, $\nu =\{x_1,x_2,\ldots ,x_i,\ldots \}$, taken by the physical subunits of the chain, see Fig. 1.

The chain has an associated *reading* direction, which is the one that makes the information along the chain meaningful (from left to right in Fig. 1), according to the language rules^33^. For example, a DNA template strand is copied (transcribed) from its 3’ end to its 5’ end during replication (transcription) and English language or sheet music are interpreted from left to right. The reading direction, which is characterized by order index *i*, imposes naturally a memory among (non-Markovian dependence on) the neighboring subunits, which restricts interactions to only one side of each subunit (left part of each current position *i*; i.e. neighbors *j*, such that $j<i$, in Fig. 1). This is in contrast to general, one-dimensional chains, in which physical interactions take place on neighbors at both sides of each subunit. In a general information setup, sequence index *i* might not be necessarily related either to a temporal order followed to set the symbolic values in the chain or to the spatial arrangement of the symbols in the physical system.

The construction of the chain is subjected to a protocol, $\lambda$, which stochastically drives its growth through privileged sequences. Information systems entail external mechanisms acting on the physical chain, which are specific of the device participating in the protocol. For example, DNA replication and transcription involves mechano-chemical forces performed by DNA/RNA polymerases (acting as the device head in Fig. 1) and artificial systems may use optical burning or magnetic setting of the characters on a tape. In the following, we will focus on the management of the sequence of symbols to find universal relations inherent to all information devices, i.e., without reference to the details of the device. Such details can be appropriately modeled for a complete thermodynamic analysis of information management in a specific system.

A general information protocol, then, combines two main actions: *writing*, which entails the incorporation of characters along the reading direction, and *revision*, which comprises the *proofreading* + *editing* of the characters^33^. More in depth, writing involves testing characters within the alphabet at each chain subunit in turn, allowing one to equilibrate with respect to the previously incorporated neighbors, and then moving on, freezing that character permanently; and revision entails a global equilibration of the chain, during which characters can fluctuate non-sequentially (within the alphabet values) at each chain subunit. As studied earlier, writing is described by a directional, stochastic chain with memory, whereas revision entails a standard equilibrium description of the full chain^34,35^. In line with this analysis, we will use superindex $\lambda =D$ for thermodynamic functions associated to the writing protocol and no superindex when referring to the proofreading and editing of the information.

We next provide four fluctuations equalities for chain-like systems that evolve irreversibly from an intial information segment. When the energy of the chain is exactly specified, the entropy change between two revised growth states, $\Delta S$, is related to the entropy change of the chains generated by writing, $\Delta S_{\nu }^{(D)}$, as follows:

1 $$\begin{aligned} \left\langle \exp \left( \Delta S_{\nu }^{(D)}/k \right) \right\rangle _D = \exp \left( \Delta S / k \right) , \end{aligned}$$

where the expected value is taken over the ensemble of sequences produced under the writing protocol ($\lambda =D$).

In this regard, the difference between the entropy change of a written chain, $\Delta S_{\nu }^{(D)}$, and that of its revised version, $\Delta S$, is related to the ratio of the writing (forward) and resetting (reverse) probabilities as follows:

2 $$\begin{aligned} \frac{p_F \left( \nu \right) }{p_R\left( \nu \right) } = \exp \left[ \left( \Delta S - \Delta S_{\nu }^{(D)} \right) / k \right] . \end{aligned}$$

Using Jensen’s inequality (the exponential is a convex function) and $\left\langle \Delta S_{\nu }^{(D)} \right\rangle _D \equiv \Delta S^{(D)}$, it follows from Eq. (1) that

3 $$\begin{aligned} \Delta S - \Delta S^{(D)} \ge 0 , \end{aligned}$$

which involves that when the chain can only exchange a defined energy with the environment, the information in the ensemble-average written chain is always greater than that in the revised chain. More in depth, by introducing the mutual information, $I\equiv -\Delta S$ and $I^{(D)} \equiv -\Delta S^{(D)}$, in the way done earlier^36^, this inequality imposes that $I \le I^{(D)}$. If the initial state were the same, independently of the protocol, then $S\ge S^{(D)}$, which indicates that the entropy of the revised chain is larger than that of the ensemble-average written chain (lower fidelity in a copying process, for example). This consequence is reminiscent of the extended version of the Clausius theorem, as we will analyze further in this paper.

**Figure 2:**
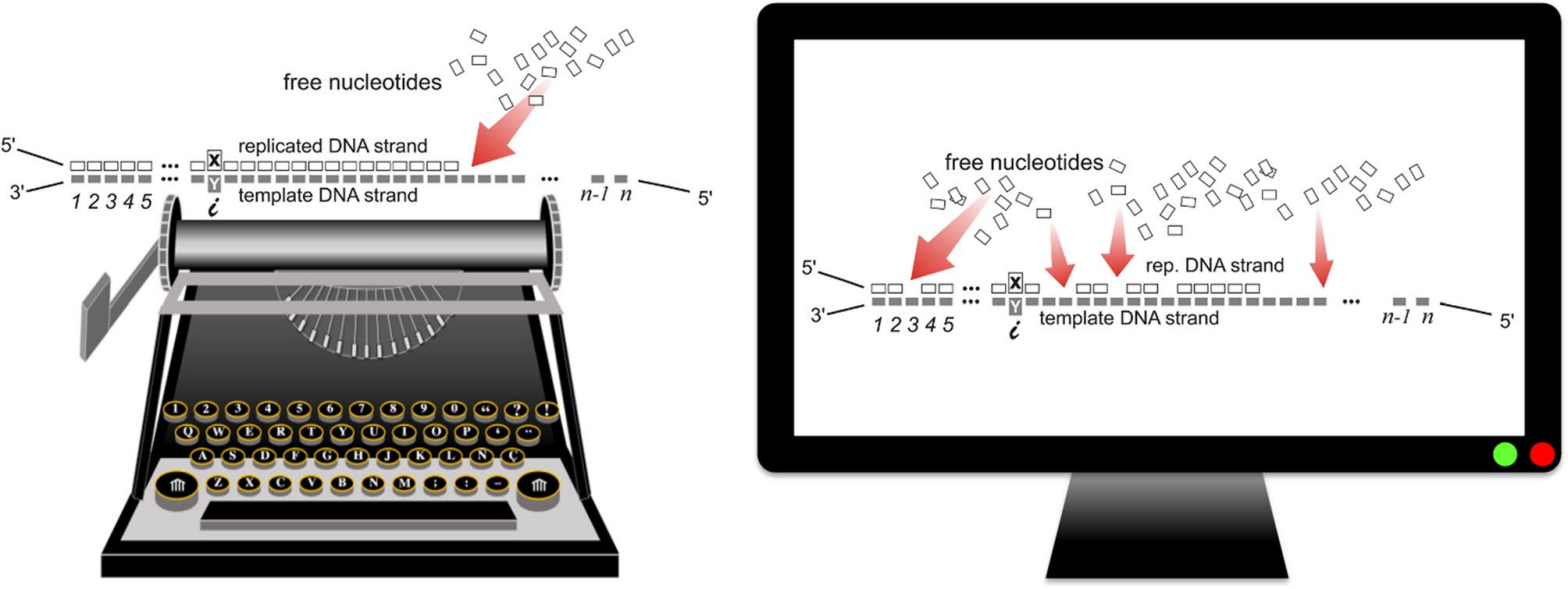
Analogy for the writing and revision statistics. The image on the left corresponds to the generation of a sequence of characters according to the directional statistics (writing): following the reading direction (from left to right in, for example, English language and sheet music, or from the 3’ end to the 5’ end of a DNA template strand during its replication), the characters are incorporated one by one, without backtracking or deletions; although errors may be detectable are not editable in a typewriting machine. The image on the right corresponds to the standard statistics (revision): the information is again interpreted according to the reading direction but now it is possible to revise it—a process that includes error detection (proofreading) and correction (editing) in a computer with a word processor—before it is printed out.

It is important to note that the expected value of a writing thermodynamic function ($S^{(D)}$, among others used below) must be interpreted as the ensemble-average value of that quantity over a sufficiently large number of printed chains in this context. In contrast, the expected value of a thermodynamic function generated after revision (*S* used so far) is equivalent to that of the chain printed once the proofreading and the editing have been applied. For the sake of clarity, we use the next analogy (Fig. 2): the writing expected value of a thermodynamic potential corresponds to the ensemble-average taken over the replicas of a text printed with a typewriting machine, whereas the revision expected value corresponds to the value of that quantity over the text generated by using a word processor on a computer, where one can proofread and edit the text as many times as desired before it is printed out.

The above theorems arise from microcanonical assumptions (Supplementary Information). This description is too limited when the information chain can indeed exchange arbitrary energies with the environment. More importantly, allowing to break this energetic limitation enables revision as an effective process (i.e. capable of decreasing the entropy of the chains generated by writing), as we analyze next. When the chain-like system is in contact with a thermal reservoir at temperature *T*, we find from canonical assumptions:

4 $$\begin{aligned} \left\langle \exp \left( \frac{\Delta S_{\nu }^{(D)}}{k} - \frac{\Delta E_{\nu }}{kT}\right) \right\rangle _D = \exp \left( \frac{\Delta S}{k} - \frac{\Delta U}{kT} \right) , \end{aligned}$$

where $\Delta E_{\nu }$ is the energy change between the initial and final microstates of a chain with sequence $\nu$ and $\Delta U$ is the energy change between the corresponding revised initial and final states.

The difference between the entropy change of a written chain and that of its revised version, given the difference between the energy change of that chain relative to its revised version, is related to the ratio of the writing and resetting probabilities at temperature *T* as follows:

5 $$\begin{aligned} \frac{P_F \left( \nu \right) }{P_R\left( \nu \right) } = \exp \left( \frac{\Delta S - \Delta S_{\nu }^{(D)}}{k} - \frac{\Delta U - \Delta E_{\nu }}{kT}\right) . \end{aligned}$$

In line with Eq. (3), the corresponding inequality for information chains in contact with a thermal reservoir is obtained by applying Jensen’s inequality to Eq. (4), namely: $T\left\langle \Delta S_{\nu }^{(D)} \right\rangle _D - \left\langle \Delta E_{\nu } \right\rangle _D \le T\Delta S-\Delta U$, in other words,

6 $$\begin{aligned} \Delta S - \Delta S^{(D)} \ge \frac{1}{T} \left( \Delta U- \Delta U^{(D)} \right) , \end{aligned}$$

with $\left\langle \Delta E_{\nu } \right\rangle _D \equiv \Delta U^{(D)}$. Using the mutual information quantity, we find $I - I^{(D)} \le \left( \Delta U^{(D)}- \Delta U \right) /T$, which allows the information of the revised chain to be higher than that of the written chain ($I>I^{(D)})$ provided that the expected energy of the revised chain is lower than that of the written chain ($\Delta U < \Delta U^{(D)}$, necessary but not sufficient condition).

Inequality (6) is reminiscent of an earlier result demonstrated independently^35^: by setting equal initial states for the written and revised chains, it is found that $S-S^{(D)} \ge \left( U-U^{(D)} \right) /T$, which was used to formulate necessary and sufficient conditions for effective proofreading and editing in information theory^33^.

It is straightforward to see that when the system is only allowed to exchange a specific energy with the environment ($\Delta E_{\nu } = \Delta U$, $\forall \nu$), we recover Eqs. (1)–(3) as direct consequences of Eqs. (4)–(6).

Theorems (1), (2) and (4), (5) are the main results of this paper, which integrate humanly-defined conceptual operations—namely, reading, writing and revision (as comprising proofreading and editing)—in information theory. In the following, we establish the notation and theoretical framework to analyze the irreversible construction of information chains, which are used to prove these results, to address thermodynamic meaning and to deduce consequences.

### Thermodynamic analysis of information chains

We study an information chain, $\nu$, as depicted in Fig. 1. Given two sequence lengths $n > m$, the microstates $\nu _n$ and $\nu _m$ (see Fig. 1), with respective configurations

7 $$\begin{aligned} \nu _{n}= & {} \{x_1,\ldots ,x_m,\ldots ,x_n\} \qquad \text {and} \end{aligned}$$

8 $$\begin{aligned} \nu _m= & {} \{x_1,\ldots ,x_m\}, \end{aligned}$$

share a common segment. The remainder sequence from *m* to *n* spells:

9 $$\begin{aligned} \nu _{n-m} = \{x_{m +1},\ldots ,x_{n}\}. \end{aligned}$$

The number of configurations for sequences $\nu _n$, $\nu _m$ and $\nu _{n-m}$ are $|\mathcal{X}|^n$, $|\mathcal{X}|^m$ and $|\mathcal{X}|^{n-m}$, respectively.

The initial state of the chain-like system is conformed by sequences of length *m*, constructed with an undefined protocol. More in depth, the system is considered to start isolated and with a defined energy for the first pair of fluctuation relation and in an equilibrium state conformed by sequences of length *m* for the second pair of relations. An initial microstate of the chain, $\nu _m$, is taken from such an ensemble, according to the surrounding conditions. Initial sequences are found, for example, as origins of DNA replication, gene promoters in transcription or the start codon in translation in molecular biology. Initial sequences may be absent in a general information system though.

Then, the chain evolves irreversibly to a microstate of length *n*; the final sequence thus represents a microstate, $\nu _n$, from an ensemble of written sequences. Alternatively, such microstate may belong to an ensemble of revised sequences (with a different probability). In the reversed evolution, the chain-like system starts at $\nu _n$ in equilibrium and ends at $\nu _m$ by attempting the recovery of the intial cell values of $\nu _{n-m}$, either irreversibly or reversibly.

The mathematical details of this analysis and proofs for the herein presented fluctuation equalities proceed in the Supplementary Information, where we consider thermodynamic functions (“*A*”) for the initial and final micro(states). More in depth, we evaluate potentials, on the one hand, at the single-sequence level (either $A_{\nu }$ or $A_{\nu }^{(D)}$) and, on the other hand, at the ensemble-average level (either $A \equiv \left\langle A_{\nu } \right\rangle$ computed with probability $p_{\nu }$ or $A^{(D)} \equiv \left\langle A _{\nu } \right\rangle _D$ computed with $p_{\nu }^{(D)}$), following the formalism derived elsewhere^34,35^. In this regard, the entropies for the writing and revision protocols, $S^{(D)} = -k \sum _{\nu } p_{\nu }^{(D)} \ln p_{\nu }^{(D)}$ and $S = -k \sum _{\nu } p_{\nu } \ln p_{\nu }$, respectively, are defined by using probabilities $p_{\nu }^{(D)} = \prod _i p \left( x_i | x_{i-1},\ldots ,x_1 \right)$, being $p \left( x_i | x_{i-1},\ldots ,x_1 \right)$ the probability of introducing a specific character at each writing step when the previous incorporations are frozen permanently, and $p_{\nu } = \exp \left( -\beta E_{\nu } \right) / \sum _{\mu } \exp \left( -\beta E_{\mu } \right)$, which is the canonical probability distribution for a chain of symbols that fluctuate non-sequentially until equilibrium, with $E_{\nu }$ the energy of the chain.

It is important to note that the writing protocol is supposed to be quasistatic: each character incorporation takes place in equilibrium conditions with respect to the previously incorporated characters, which remain fixed as the writing protocol proceeds. The fact that the writing protocol implies freezing each incorporated character introduces naturally the irreversibility of this process. Probabilities of writing, $p_{\nu }^{(D)}$, and revision, $p_{\nu }$, are equivalent only in the limit in which the memory effects are negligible (Independence Limit theorem)^34^.

### Thermodynamic meaning

To expand the physical interpretation of the above results, we study their coherence with more general results on stochastic thermodynamics. We next consider heat and work quantities for chain-like systems.

The reversible, microscopic heat between two microstates of the chain with definite energy, $\nu _m$ (in equilibrium) and $\nu _n$, in a monothermal evolution under protocol $\lambda$ is^37,38^:

10 $$\begin{aligned} Q^{(\lambda )}_{\nu } (m \rightarrow n) \equiv T \left( S^{(\lambda )}_{\nu } (n) - S_{\nu } (m) \right) . \end{aligned}$$

The protocol-dependent ensemble-average heat becomes:

11 $$\begin{aligned} Q^{(\lambda )} (m \rightarrow n) = \left\langle Q_{\nu }^{(\lambda )} (m \rightarrow n)\right\rangle _{\lambda } = T \left( S^{(\lambda )} (n) - S (m) \right) , \end{aligned}$$

and the equilibrium heat $Q (m \rightarrow n) = T \left( S(n) - S(m) \right)$ (Supplementary Information). We consider the heat positive when it is absorbed by the system and negative when it is released from the system.

It follows from Eqs. (3) and (11) that

12 $$\begin{aligned} \frac{1}{T} \left\langle Q_{\nu }^{(D)} \right\rangle _D = \frac{Q^{(D)} }{T} \le \frac{Q }{T} = \Delta S , \end{aligned}$$

which is the extended version of the Clausius theorem^37^ at constant temperature *T*, namely, $Q^{(D)} \le T \Delta S$; note that *Q* is the heat exchanged in a reversible process and $Q^{(D)}$ is the heat exchanged in a directional (irreversible) process. Inequality (12) sets a maximum to the the revised information in terms of the heat generated by the chain under (irreversible) writing, namely

13 $$\begin{aligned} I\le -Q^{(D)}/T. \end{aligned}$$

Like for the dissipated work, $W_{diss}^{(D)} \equiv W^{(D)} - \Delta F$ (see, for example,^39^), it is possible to define the dissipated heat as $Q_{diss}^{(D)} \equiv - \left( Q^{(D)} - T \Delta S \right)$, which better illustrates this trade-off. $Q_{diss}^{(D)}$ is the heat that cannot be spent in changing the entropy of the chain, eventually released to the environment. This definition can be used to show compatibility with the Gallavotti–Cohen fluctuation theorem^40^ in information chains (Supplementary Information). In a cycle, $Q_{diss}^{(D)}= -Q^{(D)}$, since the reversible heat is zero. The fact that the dissipated heat in a closed loop $-Q^{(D)} \ne 0$ is coherent with the fact that the information chain does not necessarily return to the initial microstate, $\nu _m$, by resetting the remainder sequence, $\nu _{n-m}$, to its exact initial values.

The above analysis considers chain-like systems that exchange heat as a trade for information with the environment, which remains at constant temperature. This is in contrast to the heat exchange fluctuation theorem^41^, which considers the irreversible evolution of two general systems put into contact from initially equilibrated, different temperatures.

We now set the reversible, microscopic work between two microstates of the chain as the Helmholtz free energy change^37,38^:

14 $$\begin{aligned} W^{(\lambda )}_{\nu } (m \rightarrow n) \equiv F^{(\lambda )}_{\nu } (n) - F_{\nu } (m). \end{aligned}$$

The protocol-dependent ensemble-average work yields:

15 $$\begin{aligned} W^{(\lambda )} (m \rightarrow n) = \left\langle W_{\nu }^{(\lambda )} (m \rightarrow n)\right\rangle _{\lambda } = F^{(\lambda )} (n) - F (m), \end{aligned}$$

The equilibrium, ensemble-average (macroscopic) work, $W (m \rightarrow n) = F (n) - F(m)$, emerges similarly (Supplementary Information). We consider the work positive when it is supplied to the system and negative when it is generated by the system.

The expansion of the Helmholtz free energy in terms of the entropy and the energy of the chain for single sequences^35^ along with the use of Eq. (14) makes evident the compatibility of Eqs. (4) and (5) with Jarzynski and Crooks theorems, respectively^39,42^. In addition, inequality (6), which can be expressed as $W^{(D)} \ge \Delta F$ by using Eq. (15), is coherent with Jarzynski equality after applying Jensen’s theorem, thus stating that the ensemble-average work to build information chains always supersedes the equilibrium free energy difference. In other words,

16 $$\begin{aligned} I \le \left( W^{(D)}-\Delta U \right) /T, \end{aligned}$$

which sets a maximum to the revised information in terms of the (irreversible) writing work supplied to the chain. Unlike the boundary Eq. (13), in which the nature of the system limits its capacity to release heat irreversibly, if the chain can exchange any energy with the thermal reservoir, the information can be made high just by increasing the work over the chain irreversibly. Equations (4) and (16) are in agreement with earlier fluctuation generalizations^30,43^, by noting herein that *I* is the revised information in the chain and that we are just considering purely informational entropy.

## Conclusions

We have derived fluctuation relations in the build-up of information chains. To do that, we have analyzed single sequences of characters by using the principle of microscopic reversiblity and conservation laws through a general (non-Markovian) formalism. Our results support the interpretation of thermodynamic irreversibility in terms of writing and equilibrium in terms of revision, as comprising the proofreading and editing of the information. We have shown that the connection of entropy changes, which involve structural order in thermodynamics but information in communication systems, to heat and work makes our results coherent with well-known non-equilibrium theorems. Irreversibility in an information chain appears due to the unlikelihood to grow and reset through coincident sequences. In other words, energy of a written sequence of characters (forward pathway) is dissipated as a consequence of the fact that the deletion of its associated information (backward pathway) does not necessarily return those characters to their initial symbolic values.

Our results are directly exploitable in nanoscale information setups, where the thermal noise is represented by *kT*. In this regard, fidelity during replication, transcription or translation are analyzable with these theorems, where the homeostatic control on a DNA molecule, a messenger RNA or a protein in the cell presets temperature variations in a narrow interval. The application of our results to more general communication channels requires the description of noise by specific parameters^33^, other than the thermal level.

## Supplementary Information

Supplementary Information.

## Data Availability

All data generated or analysed during this study are included in this published article (and its supplementary information file).
