# Supplementary material for: Fluctuation relations for irreversible emergence of information
Source: Sci Rep. 2022 Oct 14;12:17230. doi: 10.1038/s41598-022-21729-9 (PMC9568592; doi:10.1038/s41598-022-21729-9)
Supplement: Supplementary file 1 — Supplementary Information. [file 41598_2022_21729_MOESM1_ESM.pdf]

Supplementary Information:

Fluctuation relations for irreversible emergence of information

J. Ricardo Arias-Gonzalez*

Centro de Tecnologías Físicas, Universitat Politècnica de València, Camino de Vera s/n, 46022 Valencia, Spain

*E-mail: ricardo.ariasgonzalez@upv.es, ricardo.ariasgonzalez@gmail.com

Thermodynamic analysis of information chains

We assume that the construction of the chain is not constrained to a definite time. The probability of a sequence depends on the energy of the chain $E_\nu = \sum_i E_i$, which is a sum over the energies of the chain subunits. Due to memory effects, the energy of each object depends in turn on its previous neighbors, namely, $E_i = E(x_i; x_{i-1}, \ldots, x_1)$. These energies are the result of physicochemical interactions among the subunits, which may be contributed by setup mechanisms that correlate current objects with associated previous neighbors. DNA replication (as well as transcription and translation) is a natural example: nucleotide pairs along a DNA chain base-stack onto their previous neighbors releasing energy [1], besides being correlated with a certain number of previous base-pairs by a polymerase protein. Either natural or ad hoc in an artificial device, these energies, in which underlying language rules may be represented, provide a feedback to channel information through the chain.

We denote by λ the protocol by which the information in the chain evolves. The reverse protocol, λ^{-1}, is characterized by the same set of parameters as λ with opposite evolution. A thermodynamic function, "A", can be averaged over the chains complying with the evolving constraints, prescribed by the protocol λ, by taking expected values, $A^{(\lambda)} \equiv \left\langle A_\nu^{(\lambda)} \right\rangle_\lambda = \sum_\nu p_\nu^{(\lambda)} A_\nu^{(\lambda)}$ [2], where $p_\nu^{(\lambda)}$ is the probability distribution according to protocol λ.

Protocols are usually stepwise and directional, as performed by a Turing machine. In such writing protocols, the device 'head' incorporates symbols, one at a time, to the tape in one direction (from left to right in Fig. 1 of main text), making the probability at each writing step express as $p\left(x_i | x_{i-1}, \ldots, x_1\right) = e^{-\beta E(x_i; x_{i-1}, \ldots, x_1)} / \sum_{x_i'} e^{-\beta E\left(x_i'; x_{i-1}, \ldots, x_1\right)}$

and the probability of a sequence as $p_\nu^{(D)} = e^{-\beta E_\nu}/Z_\nu^{(D)}$, being $Z_\nu^{(D)}$ the so-called sequence-dependent partition function [3]:

$$Z_\nu^{(D)}(\beta, n) \equiv \sum_{x'_1,\ldots,x'_n} \exp\left[-\beta \sum_{i=1}^{n} E\left(x'_i; x_{i-1}, \ldots, x_1\right)\right]. \tag{S1}$$

As explained earlier [2, 3], the standard partition function,

$$Z(\beta, n) \equiv \sum_{x_1,\ldots,x_n} \exp\left[-\beta \sum_{i=1}^{n} E\left(x_i; x_{i-1}, \ldots, x_1\right)\right], \tag{S2}$$

which defines the equilibrium probabilities $p_\nu = \exp\left(-\beta E_\nu\right)/Z$, does not restrict how to access each final configuration. This formalism comprises the concept of revision, which includes the proofreading and the editing of the information in the chain [2, 4]. Ensemble-average equilibrium thermodynamic functions are describable in a similar fashion as the directional ones, this time by taking expected values with distribution p_ν, namely, $A \equiv \langle A_\nu \rangle = \sum_\nu p_\nu A_\nu$.

Proofs of Fluctuation relations

We consider a chain-like system that is initially configured as a microstate ν_m in equilibrium; that is, ν_m belongs to an ensemble of statistically similar microstates in the absence of a protocol. Then, the chain evolves to a final microstate ν_n under writing, $\lambda = D$, see Fig. 1 in the main text. As for the backwards evolution (resetting), the chain transforms from ν_n in equilibrium to ν_m, following the inverse protocol, D^{-1}.

Chain with constrained energy changes

The system is prepared in isolation, with a defined energy both for the forward and reverse evolutions at temperature T. It then grows into a (micro)state at the same temperature by exchanging a precise energy. The writing protocol transforms the chain from an ensemble of $M \leq |\mathcal{X}|^m$ sequences with expected entropy $S(m)$ into a microstate with entropy $S_\nu^{(D)}(n)$. In the reverse evolution (resetting), the chain starts in an equilibrium defined by $N \leq |\mathcal{X}|^n$ available sequences and entropy $S(n)$, and transforms into a microstate with entropy $S_\nu^{(D^{-1})}(m)$.

We now demonstrate the first theorem in the main text, Eq. (1). The probability of the initial state in terms of the microcanonical entropy is $p_\nu(m) = 1/M = \exp\left(-S(m)/k\right)$ and the directional probability for a single trajectory $p_\nu^{(D)} = \exp\left(-S_\nu^{(D)}/k\right)$ [2, 5, 6]. The change in entropy for a chain constructed under writing is $\Delta S_\nu^{(D)}(m, n) \equiv S_\nu^{(D)}(n) - S_\nu(m)$,

where $S_\nu(m) = S(m)$ in the initial conditions described above. Then:

$$\left\langle e^{\frac{1}{k}\Delta S_\nu^{(D)}(m,n)} \right\rangle_D = \left\langle e^{\frac{1}{k}\left(S_\nu^{(D)}(n)-S_\nu(m)\right)} \right\rangle_D = \left\langle \frac{p_\nu(m)}{p_\nu^{(D)}(n)} \right\rangle_D = \sum_{\nu_n=1}^{N} p_\nu^{(D)}(n)\frac{p_\nu(m)}{p_\nu^{(D)}(n)} =$$
$$= \sum_{\nu_n=1}^{N} p_\nu(m) = \sum_{\nu_m=1}^{M} \sum_{\nu_{n-m}=1}^{N/M} p_\nu\,(m) = \frac{N}{M} = e^{\frac{1}{k}\Delta S(m,n)}, \tag{S3}$$

which yields:

$$\left\langle \exp\left(\frac{\Delta S_\nu^{(D)}}{k}\right) \right\rangle_D = \exp\left(\frac{\Delta S}{k}\right). \tag{S4}$$

We next demonstrate Eq. (2) in the main text. The transition forward probability, $p_F(m \to n)$, equals the probability $\exp\left(-S(m)/k\right)$ of starting in equilibrium times the probability $\exp\left(-S_\nu^{(D)}(n)/k\right)$ of ending in a microstate through irreversible writing. The transition reverse probability follows the same rationale, namely, $p_R(m \leftarrow n) = \exp\left(-S(n)/k\right) \times \exp\left(-S_\nu^{(D^{-1})}(m)/k\right)$. Then, the ratio of forward and reverse probabilities read

$$\frac{p_F(m \to n)}{p_R(m \leftarrow n)} = \exp\left(\frac{S(n)-S(m)}{k}\right)\exp\left(-\frac{S_\nu^{(D)}(n)-S_\nu^{(D^{-1})}(m)}{k}\right), \tag{S5}$$

where $S_\nu^{(D^{-1})}(m) = S_\nu^{(D)}(m)$, given that we start at the final microstate and recover the initial one by removing objects (or by resetting them to their initial values) in sequence ν_{n-m}. Using $\Delta S = S(n) - S(m)$ and $\Delta S_\nu^{(D)} = S_\nu^{(D)}(n) - S_\nu^{(D)}(m)$, we find:

$$\frac{p_F(m \to n)}{p_R(m \leftarrow n)} = \exp\left(\frac{\Delta S(m,n) - \Delta S_\nu^{(D)}(m,n)}{k}\right), \tag{S6}$$

which can be expressed as:

$$\frac{p_F\,(\nu)}{p_R\,(\nu)} = \exp\left[\left(\Delta S - \Delta S_\nu^{(D)}\right)/k\right]. \tag{S7}$$

It is straightforward to see that Eq. (S4) (i.e. Eq. (1) in the main text) can also be obtained from Eq. (S7) (Eq. (2) in the main text). Certainly, by summing over all sequences on both sides of Eq. (S7), we find:

$$\left\langle e^{\frac{1}{k}\Delta S_\nu^{(D)}} \right\rangle_D = \sum_\nu p_F\,(\nu)\, e^{\frac{1}{k}\Delta S_\nu^{(D)}} = e^{\frac{1}{k}\Delta S}\sum_\nu p_R\,(\nu) = e^{\frac{1}{k}\Delta S}. \tag{S8}$$

Chain in contact with a thermal reservoir

The system starts in equilibrium with a thermal bath at temperature T, with which it can exchange energy at all times. The Helmholtz free energy characterizes the equilibrium initial states in these conditions, both for the forward and the reverse evolutions. The change in free energy for a chain constructed under writing is $\Delta F_\nu^{(D)}(m,n) \equiv F_\nu^{(D)}(n) - F_\nu(m)$, with $F_\nu(m) = -kT \ln Z(m) = F(m)$, independent of the sequence [2, 5]. Then:

$$\begin{aligned}\left\langle e^{-\beta \Delta F_\nu^{(D)}(m,n)} \right\rangle_D = \left\langle e^{-\beta\left(F_\nu^{(D)}(n) - F_\nu(m)\right)} \right\rangle_D &= \left\langle \frac{Z_\nu^{(D)}(n)}{Z(m)} \right\rangle_D \\ &= \frac{Z(n)}{Z(m)} = e^{-\beta \Delta F(m,n)}, \end{aligned} \tag{S9}$$

being $\Delta F(m,n) = F(n) - F(m)$ the equilibrium free energy change and $\left\langle Z_\nu^{(D)}(n) \right\rangle_D = Z(n)$, as demonstrated elsewhere [2, 3]. The Helmholtz free energy can be expressed in terms of the energy and the entropy, both for single sequences and equilibrium ensemble averages, hence transforming Eq. (S9) into

$$\left\langle \exp\left(\frac{\Delta S_\nu^{(D)}}{k} - \frac{\Delta E_\nu}{kT} \right) \right\rangle_D = \exp\left(\frac{\Delta S}{k} - \frac{\Delta U}{kT} \right), \tag{S10}$$

where $\Delta E_\nu(m,n) = E_\nu(n) - E_\nu(m)$ is the energy change between the initial and final sequences and $\Delta U(m,n) = U(m) - U(n)$ the corresponding expected energy change in equilibrium conditions. Equation (S10) closes the proof to Eq. (4) in the main text.

Next, we derive Eq. (5) in the main text. The transition forward canonical probability, $P_F(m \to n)$, equals the probability $p_\nu(m)$ of starting in equilibrium times the probability $p_\nu^{(D)}(n)$ of ending in a general microstate. The transition reverse canonical probability follows the same argument, namely, $P_R(m \leftarrow n) = p_\nu(n) \times p_\nu^{(D^{-1})}(m)$. Then, the ratio of forward and reverse probabilities reads:

$$\begin{aligned}\frac{P_F(m \to n)}{P_R(m \leftarrow n)} &= \frac{p_\nu(m) \times p_\nu^{(D)}(n)}{p_\nu(n) \times p_\nu^{(D^{-1})}(m)} = \\ &= \frac{\exp\left(-\beta E_\nu(m)\right)}{Z(m)} \frac{\exp\left(-\beta E_\nu(n)\right)}{Z_\nu^{(D)}(n)} \times \left(\frac{\exp\left(-\beta E_\nu(n)\right)}{Z(n)} \frac{\exp\left(-\beta E_\nu(m)\right)}{Z_\nu^{(D^{-1})}(m)} \right)^{-1} \\ &= \frac{Z(n)}{Z(m)} \frac{Z_\nu^{(D^{-1})}(m)}{Z_\nu^{(D)}(n)}. \end{aligned} \tag{S11}$$

The partition functions in Eq. (S11) can be expressed in terms of the free energies,

$$
\begin{aligned}
Z(n) &= \exp\left(-\beta F(n)\right), &\text{(S12)}\\
Z_\nu^{(D)}(n) &= \exp\left(-\beta F_\nu^{(D)}(n)\right); &\text{(S13)}\\
Z(m) &= \exp\left(-\beta F(m)\right), &\text{(S14)}\\
Z_\nu^{(D^{-1})}(m) &= \exp\left(-\beta F_\nu^{(D^{-1})}(m)\right), &\text{(S15)}
\end{aligned}
$$

where $F_\nu^{(D^{-1})}(m) = F_\nu^{(D)}(m)$, given that we start at the final microstate and recover the initial one by removing objects (or by resetting them to their initial values) in sequence ν_{n-m}. Then

$$\frac{P_F(m \to n)}{P_R(m \leftarrow n)} = \exp\left(\frac{\Delta F_\nu^{(D)}(m,n) - \Delta F(m,n)}{kT}\right), \tag{S16}$$

where we have used that $\Delta F(m,n) = F(n) - F(m)$. Within the canonical formalism, this result can also be expressed in terms of the entropy [2], more significant in information theory:

$$\frac{P_F(\nu)}{P_R(\nu)} = \exp\left(\frac{\Delta S - \Delta S_\nu^{(D)}}{k} - \frac{\Delta U - \Delta E_\nu}{kT}\right). \tag{S17}$$

It is straightforward to see that Eq. (4) in the main text can also be obtained from Eq. (5) in the main text, it is sufficient to show herein that Eq. (S9) emerges from Eq. (S16). Certainly, by summing over all sequences on both sides of Eq. (S16), we find:

$$\left\langle e^{-\beta \Delta F_\nu^{(D)}}\right\rangle_D = \sum_\nu P_F(\nu)\, e^{-\beta \Delta F_\nu^{(D)}} = e^{-\beta \Delta F} \sum_\nu P_R(\nu) = e^{-\beta \Delta F}. \tag{S18}$$

Expected Work and Heat

The expression for the protocol-dependent ensemble-average work (Eq. (14) in the main text) is:

$$
\begin{aligned}
W^{(\lambda)}(m \to n) &= \left\langle W_\nu^{(\lambda)}(m \to n)\right\rangle_\lambda = \sum_\nu p_\nu^{(\lambda)}\left[F_\nu^{(\lambda)}(n) - F_\nu(m)\right]\\
&= \sum_{\nu_n=1}^{N} p_\nu^{(\lambda)}(n) F_\nu^{(\lambda)}(n) - \sum_{\nu_n=1}^{N} p_\nu^{(\lambda)}(n) F(m)\\
&= \left\langle F_\nu^{(\lambda)}(n)\right\rangle_\lambda - F(m) = F^{(\lambda)}(n) - F(m), \qquad\text{(S19)}
\end{aligned}
$$

where we have used that the equilibrium Helmholtz free energy is independent of the sequence, as shown above. The corresponding equilibrium work (see the main text) follows

the same reasoning by adding that $F_\nu(n) = -kT \ln Z(n) = F(n)$. Then $W(m \to n) = \sum_\nu p_\nu \left[F_\nu(n) - F_\nu(m)\right] = F(n) - F(m)$.

Concerning the expresión for the protocol-dependent heat (Eq. (11) in the main text), since the system is initially prepared in isolation, the entropy just depends on the number of available configurations, not on the precise sequence, $S_\nu(m) = S(m)$. Then

$$Q^{(\lambda)}(m \to n) = T\left(S^{(\lambda)}(n) - S(m)\right) \tag{S20}$$

appears similarly as $W^{(\lambda)}(m \to n)$ by replacing F for TS, with T a constant. The corresponding equilibrium heat is immediate for initial and final isolated conditions, $Q(m \to n) = T\left(S(n) - S(m)\right)$.

Gallavotti-Cohen theorem for information chains

We introduce the rate, $\sigma^{(\lambda)}$, at which the chain exchanges heat with the bath under a certain protocol λ from the dissipated heat $Q^{(\lambda)}_{diss}$ (see the main text):

$$\sigma^{(\lambda)} \equiv \frac{1}{T\Delta t} Q^{(\lambda)}_{diss} = -\frac{Q^{(\lambda)} - Q}{T\Delta t} = -\frac{\Delta S^{(\lambda)} - \Delta S}{\Delta t}, \tag{S21}$$

where Δt is a time interval. With this definition, it is straightforward to derive the Gallavotti-Cohen expression [7], emerging from the so-called *Fluctuation Theorem* [8, 9], for an information chain that is physical. Gallavotti-Cohen equality connects the forward and reverse heat-rate probability distributions for steady-state, non-equilibrium processes. It appears naturally within our formalism by following a similar argument as the one above for Eq. (S6); certainly, by using Eq. (S21) in the derivation of Eq. (S6), it follows that

$$\lim_{\Delta t \to \infty} \frac{k}{\Delta t} \ln\left(\frac{p_f}{p_r}\right) = \sigma^{(D)}, \tag{S22}$$

where

$$p_f = \exp\left(-S(m)/k\right) \times \exp\left(-S^{(D)}(n)/k\right) \quad \text{and} \tag{S23}$$

$$p_r = \exp\left(-S(n)/k\right) \times \exp\left(-S^{(D^{-1})}(m)/k\right). \tag{S24}$$

Here, $\exp\left(-S^{(D)}(n)/k\right)$ and $\exp\left(-S^{(D^{-1})}(m)/k\right)$ are the probabilities of finding the chain with steady-state energies $U^{(D)}(n)$ and $U^{(D^{-1})}(m)$, respectively. In a cycle, $Q^{(D)}_{diss} = -Q^{(D)}$ and $\sigma^{(D)} = -Q^{(D)}/T\Delta t$, since the reversible heat is zero; the internal energy change of the chain is $U^{(D)}(n) - U^{(D^{-1})}(m)$. As explained in the main text, the fact that $\sigma^{(D)} = -Q^{(D)}/T\Delta t$ in a closed loop can also be understood from the fact that the chain does not necessarily recover the initial sequence, ν_m, by recovering the microstate of the remainder sequence, ν_{n-m}.

Supplementary References

[1] J. SantaLucia, Jr. and D. Hicks, *The Thermodynamics of DNA Structural Motifs*, Annu. Rev. Biophys. Biomol. Struct. **33**, 415–440 (2004).

[2] J. R. Arias-Gonzalez, *Thermodynamic framework for information in nanoscale systems with memory*, J. Chem. Phys. **147**, 205101 (2017).

[3] J. R. Arias-Gonzalez, *Information management in DNA replication modeled by directional, stochastic chains with memory*, J. Chem. Phys. **145**, 185103 (2016).

[4] J. R. Arias-Gonzalez, *Writing, Proofreading and Editing in Information Theory*, Entropy **20**, 368 (2018).

[5] J. R. Arias-Gonzalez, *Microscopically Reversible Pathways with Memory*, Mathematics **9**, 127 (2021).

[6] U. Seifert, *Entropy production along a stochastic trajectory and an integral fluctuation theorem*, Phys. Rev. Lett. **95**, 040602 (2005).

[7] G. Gallavotti and E. G. D. Cohen, *Dynamical Ensembles in Nonequilibrium Statistical Mechanics*, Phys. Rev. Lett. **74**, 2694 (1995).

[8] D. J. Evans and D. J. Searles, *Equilibrium microstates which generate second law violating steady states*, Phys. Rev. E **50**, 1645 (1994).

[9] D. J. Evans, E. G. D. Cohen and G. P. Morriss, *Probability of Second Law Violations in Shearing Steady States*, Phys. Rev. Lett. **71**, 2401 (1993).
